# Blackberry Feeding Increases Fat Oxidation and Improves Insulin Sensitivity in Overweight and Obese Males

**DOI:** 10.3390/nu10081048

**Published:** 2018-08-09

**Authors:** Patrick M. Solverson, William V. Rumpler, Jayme L. Leger, Benjamin W. Redan, Mario G. Ferruzzi, David J. Baer, Thomas W. Castonguay, Janet A. Novotny

**Affiliations:** 1 USDA, ARS, Beltsville Human Nutrition Research Center, Beltsville, MD 20705, USA; Patrick.Solverson@ars.usda.gov (P.M.S.); Bill.Rumpler@ars.usda.gov (W.V.R.); Jayme.Leger@gmail.com (J.L.L.); David.Baer@ars.usda.gov (D.J.B.); 2 Nutrition and Food Science, University of Maryland, College Park, MD 20742, USA; twc@umd.edu; 3 Interdepartmental Nutrition Program, Purdue University, West Lafayette, IN 47906, USA; Benjamin.Redan@fda.hhs.gov; 4 Plants for Human Health Institute, North Carolina State University, Kannapolis, NC 28081, USA; mferruz@ncsu.edu

**Keywords:** berries, indirect calorimetry, overweight, obesity, fat oxidation, glucose, insulin sensitivity, anthocyanins, flavonoids, polyphenols

## Abstract

Berries and other anthocyanin-rich treatments have prevented weight gain and adiposity in rodent models of diet-induced obesity. Their efficacy may be explained by modulation of energy substrate utilization. However, this effect has never been translated to humans. The objective of this study was to evaluate the effects of berry intake on energy substrate use and glucoregulation in volunteers consuming a high-fat diet. Twenty-seven overweight or obese men were enrolled in a randomized, placebo-controlled crossover study with two treatment periods. Subjects were fed an investigator controlled, high-fat (40% of energy from fat) diet which contained either 600 g/day blackberries (BB, 1500 mg/day flavonoids) or a calorie and carbohydrate matched amount of gelatin (GEL, flavonoid-free control) for seven days prior to a meal-based glucose tolerance test (MTT) in combination with a 24 h stay in a room-sized indirect calorimeter. The washout period that separated the treatment periods was also seven days. The BB treatment resulted in a significant reduction in average 24 h respiratory quotient (RQ) (0.810 vs. 0.817, BB vs. GEL, *p* = 0.040), indicating increased fat oxidation. RQ during the MTT was significantly lower with the BB treatment (0.84) compared to GEL control (0.85), *p* = 0.004. A 4 h time isolation during dinner showed similar treatment effects, where RQ was reduced and fat oxidation increased with BB (0.818 vs. 0.836, 28 vs. 25 g, respectively; BB vs. GEL treatments). The glucose AUC was not different between the BB and GEL treatments during the MTT (3488 vs. 4070 mg·min/dL, respectively, *p* = 0.12). However, the insulin AUC was significantly lower with the BB compared to the GEL control (6485 vs. 8245 µU·min/mL, *p* = 0.0002), and HOMA-IR improved with BB (*p* = 0.0318). Blackberry consumption may promote increased fat oxidation and improved insulin sensitivity in overweight or obese males fed a high fat diet.

## 1. Introduction

Two-thirds of American adults can be classified as either overweight or obese [1]. Obesity leads to an increased risk for cardiovascular disease, diabetes, cancer, and other morbidities; it also burdens our health care system at a level of $147 billion annually [2]. Lifestyle modification remains an attractive strategy for combating obesity, as dietary approaches offer solutions to weight loss and improved health without negative side effects. To date, medical and pharmacological approaches to obesity treatment have failed to produce an alternative to improved/informed diet choices and increased physical activity [3]. Within the realm of diet choices, the protective effects of fruit and vegetable consumption against all-cause mortality and cardiovascular disease are notable [4,5], and these effects may be related to their polyphenol content. As reported in the Predimed study, there is a protective effect of higher intakes of polyphenol-rich foods against all-cause mortality [6]. Anthocyanins are one of the main classes of flavonoids (a type of polyphenol) garnering substantial attention in regard to prevention of obesity and related morbidities. Anthocyanins are responsible for the red, blue, and purple hues of fruits and vegetables. Of the fruits and vegetables found in a typical American diet, berries provide one of the greatest sources for anthocyanin consumption [7]. 

A common dietary anthocyanin, cyanidin-3-*O*-β-glucoside (C3G), was demonstrated to potentially influence obesity risk when delivered in the form of purple corn color. Tsuda et al. [8] fed mice a high fat (60% energy from fat) diet for twelve weeks supplemented with or without purple corn color that provided 2–4 mg of C3G per day. Mice fed C3G had reduced body weight, adipocyte size, and reduced expression of lipogenic enzymes in hepatic and adipose tissues [8]. These findings were followed by a number of berry anthocyanin research studies in rodent models of obesity with mixed, but mostly positive effects [9,10,11,12,13,14,15,16]. Several of these studies have also reported improvements in insulin sensitivity [10,14,15,16].

To facilitate translation of preclinical findings to humans, we used indirect calorimetry to test the hypothesis that berries would influence substrate trafficking in humans and to give clues to the potential of anthocyanin-rich foods to reduce obesity. Blackberries were chosen as the anthocyanin source due to their high content of cyanidin 3-glucoside, an especially common anthocyanin in the food supply. In addition, potential glucoregulatory effects were also investigated. The objective of this study was to evaluate the effects of berry intake on energy substrate use and glucoregulation in volunteers consuming a high-fat diet.

## 2. Materials and Methods 

Subjects were recruited from the Washington D.C. metropolitan area via email advertisements. Eligibility was determined by review of a health history questionnaire in addition to blood and urine clinical chemistries. Only males were recruited so as to avoid added variability in calorimetry measures due to women’s menstrual cycles [17]. Overweight/obese men (BMI > 25 kg/m^2^) were recruited as a population with potential to benefit from anti-obesity effects of blackberries. Volunteers were excluded from participation if they had gastrointestinal or metabolic diseases or disorders, type-2 diabetes under pharmaceutical treatment, fasting glucose ≥126 mg/dL, fasting triglycerides ≥300 mg/dL, use of anti-obesity medication/supplements for the preceding 6 months, cardiovascular disease, use of tobacco products, use of antibiotics in the preceding month, use of polyphenol-rich supplements, dietary pattern of high polyphenol intake, or adverse reactions to blackberries. Volunteers were excluded if they had a weight change of greater than 10% in the past 12 months. The study protocol (Medstar IRB project # 2013-037) was approved by Medstar Health Research Institute institutional review board (Hyattsville, MD, USA), and all subjects provided written, informed consent. The study was conducted at the USDA Beltsville Human Nutrition Research Center in Beltsville, MD, USA. Clinical trial registry number: This trial was registered at www.clinicaltrials.gov (NCT01932879). 

### 2.1. Study Design

The study was a randomized, placebo controlled, cross-over with two treatments. Participants were not blinded to the treatments due to difficulty in blinding to food treatments. Participants consumed either 600 g of whole blackberries (BBs) (frozen, removed from freezer within 12 h of consumption) or a calorically matched amount of artificially flavored gelatin (GEL) daily for one week as part of a fully controlled diet. Frozen blackberries were purchased in bulk as one lot from Sysco (Jessup, MD, USA) and stored at −20 °C. Gelatin was prepared from a commercially available powdered mix (Kraft Jell-O brand, strawberry, Chicago, IL, USA). Treatment periods were separated by a one-week washout during which participants were instructed to avoid red and purple pigmented fruits and vegetables. Treatment doses were divided in two equal sized portions of 300 g each and consumed at breakfast and dinner. In an effort to maximize treatment differences, the subjects consumed the blackberries at twice the amount of fruit intake recommended in the 2015–2020 Dietary Guidelines for Americans (600 g of blackberries is approximately 4 cups) [18]. The blackberry treatment provided ~1476 mg of flavonoids and ~361 mg of total anthocyanins daily as analyzed using a published LC/MS method (Table 1) [19]. Participants were randomly assigned to one of the two treatment orders by the study coordinator, and treatments were color coded to conceal treatment identities from staff.

### 2.2. Diets

Participants were fed a controlled diet with a macronutrient profile of 40% of energy from fat, 45% from carbohydrate, and 15% from protein; the diet was devoid of anthocyanins except for the blackberry treatment. Food intake was scaled according to energy needs for each participant based on the Harris–Benedict equation and an activity factor. Meals were provided as a 2-day menu rotation using common American menu items. Participants were instructed to eat only the food provided to them by the Nutrition Center. Monday through Friday, participants were required to eat both breakfast and dinner at the Nutrition Center, while both lunch and weekend meals were packed for consumption off-site. Diet adherence was assessed by monitoring changes to body weight. To encourage compliance, subjects were under observation by dietetic technicians when consuming breakfast and dinner meals at the Nutrition Center.

### 2.3. Assessments Sample Collection and Analysis

The final twenty-four hours of each diet period was completed with participants residing in a room-sized indirect calorimetry chamber [20] for measurement of respiratory gasses and subsequent calculation of respiratory quotient (RQ), energy expenditure, and substrate oxidation. Participants arrived at the center between 4:00 p.m. and 5:00 p.m. for their 24-h chamber stay. Participants were instructed to engage only in quiet activities with the exception of thirty minutes of scheduled, supervised treadmill walking (treadmill programmed for 3 mph for 30 min) in the early afternoon of the final day of each calorimeter session. Meals and bottled water were delivered through an air lock, and each chamber had a ceiling fan set to low to promote even mixing of respiratory gases. 

Chamber air was sampled every 100 s, and gas concentrations were measured simultaneously with a Perkin Elmer MGA 1200 multiple gas analyzer (Waltham, MA, USA). Prior to the subjects’ stay, each chamber was calibrated via ethanol combustion. A deconvolution algorithm [21] was applied to gas data to reduce instrumental noise and to generate minute-by-minute estimates for CO_2_ production and O_2_ consumption. RQ, energy expenditure, and substrate oxidation were calculated for the 24-h period, as well as for 5 time isolations: Evening—4 h starting with the first bite of dinner, ~6:00 p.m. to 10:00 p.m.; Nighttime—two hours at night (between 2:00 a.m. and 4:00 a.m.), which would provide an estimate of resting metabolic rate; Morning—4 h starting with the first bite of breakfast, ~7:00 a.m.–11:00 a.m.; Afternoon—2 h starting with the first bite of lunch, ~12:00 p.m.–2:00 p.m.; Exercise—30 min during treadmill walking (2:00 p.m.–2:30 p.m.).

In order to determine modulation of insulin sensitivity and glucose tolerance by blackberries, a meal-based oral glucose tolerance test (MTT) was administered on the morning of day 7 while the participants were completing their 24-h calorimeter stay. Participants were awakened at approximately 6:00 a.m., and an indwelling catheter was placed in the antecubital vein, after which 2 blood draws were collected before consumption of their meal. Participants were able to extend their arm to the outside of the chamber by way of a small arm port that was only opened for catheter setting and subsequent blood collections. Subjects were then provided with approximately 75 g of carbohydrate in the form of toaster waffles and syrup in combination with either 302 g of blackberries or 273 g of gelatin closely matched for carbohydrate and energy (Table 2**)**.

Participants had ten minutes to consume both the waffle meal and their respective treatment foods. Subsequent blood sampling was collected at 30, 60, 90, 120, 180, and 240 min after the first bite of breakfast. Blood was collected in serum, plasma-EDTA, and sodium-fluoride tubes, centrifuged, aliquoted, and stored at −80 °C until analyzed. Serum glucose, non-esterified fatty acids (NEFAs), and triglycerides were analyzed using standard protocols on an automated clinical chemistry analyzer (Vitros 5,1 FS, www.orthoclinical.com). Insulin was measured using ELISA kits (EMD Millipore, Burlington, MA, USA, inter-assay CV = 2.83%) per the manufacturer’s instructions on an automated plating and spectrophotometry system (DSX workstation, Dynex Technologies, Chantilly, VA, USA). 

### 2.4. Calculations and Statistics 

Due to lack of data on the effect of polyphenols on fat oxidation, this study was conducted as a pilot study. The target enrollment was *n* = 16 based on previous calorimetry studies at the Beltsville Human Nutrition Research Center [22]. Additional subjects were enrolled due to weather related electrical disruptions in data collection. Each calorimetry outcome parameter (averaged RQ and summed fat oxidation, carbohydrate oxidation, and energy expenditure) was calculated for each of the time isolations (24 h, evening, nighttime, morning, afternoon, and exercise). Energy expenditure was calculated using the Weir equation, and substrate oxidation (g of fat or carbohydrate used for energy) was calculated using the equations of Livesey and Elia [23]. Because the participants were weight stable and on a controlled protein intake that was the same for both feeding periods, they were assumed to be in nitrogen balance. Thus, the rate of protein oxidation (required to calculate oxidation of fat and carbohydrate with Livesey equations) was determined using protein intake from the controlled feeding menu. MTT incremental area under the curve (iAUC) was calculated for glucose and insulin, and area under the curve (AUC) for NEFAs using central Riemann-sum. Fasting concentrations of glucose and insulin were used to calculate homeostasis model assessment of insulin resistance and β-cell function (HOMA-IR and HOMA-B) as follows: HOMA-IR = (fasting serum insulin × fasting serum glucose)/22.5 and HOMA-B = (20 × fasting serum insulin)/(fasting serum glucose − 3.5), where fasting serum insulin concentrations were expressed as µU insulin/mL serum and fasting serum glucose concentrations were expressed as mmol glucose/L serum [24]. 

Linear mixed models were used to test for statistically significant differences between the blackberry and gelatin treatments using “proc mixed” repeated measures analysis of covariance with SAS version 9.4 (SAS institute, Cary, NC, USA). Normality and homoscedasticity of residuals were determined by the Shapiro–Wilk test and visual inspection of residual plots, respectively. Non-normality of residuals was addressed by mathematical transformation. Model estimates of response variables from each treatment were repeated on subject fit with the best covariance structure, which was determined by information criteria as well as visual inspection of residual plots for each covariance structure used in preliminary analyses. The main effect of treatment and covariates of subject BMI, age, and sequence order were included in the model statement. Interactions of BMI and age with treatment were determined with backward elimination of non-significant terms. Random side error effects were estimated for subjects nested in sequence. Data are presented as LSmeans for each treatment and *p* < 0.05 was considered statistically significant.

## 3. Results

**Figure 1:**
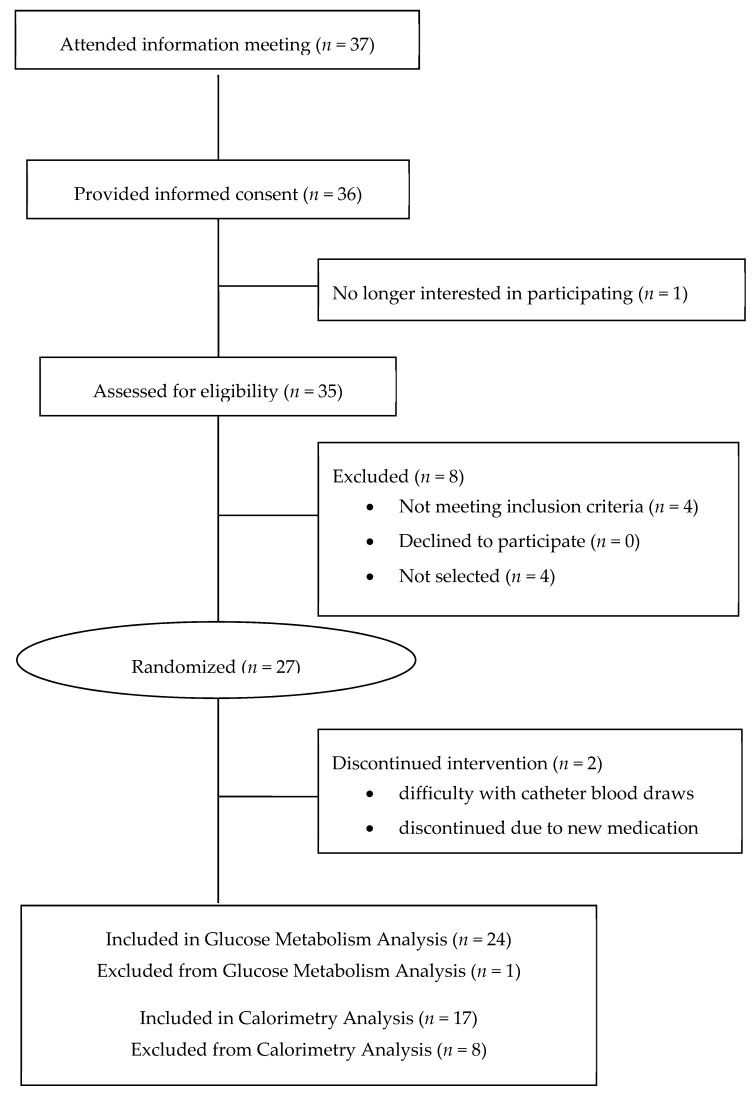
CONSORT (Consolidated Standards of Reporting Trials) diagram for the blackberry calorimetry study. One subject was excluded from the meal tolerance analysis for noncompliance. Eight subjects were excluded from the calorimetry dataset due to instrument failure during data collection.

Thirty-six potential volunteers provided informed consent, 35 of which completed the screening, and 27 of which were selected for participation in the study (Figure 1). Two volunteers withdrew from the study, one because of perceived discomfort of the intravenous catheter and the other due to prescribed steroid use for a sinus infection during the intervention. A total of 17 subjects had successful calorimetry stays that provided reliable data of the gaseous exchanges that were used in the analysis, while data for the other eight were lost due to instrument failure during weather-related power outages during data collection. Baseline subject characteristics are reported for the meal-based glucose tolerance test and calorimetry datasets in order to reflect the loss of subjects from the calorimetry dataset (Table 3). There were no significant differences in subject characteristics between the two different datasets generated in this study. 

### 3.1. Energy Expenditure

**Figure 2:**
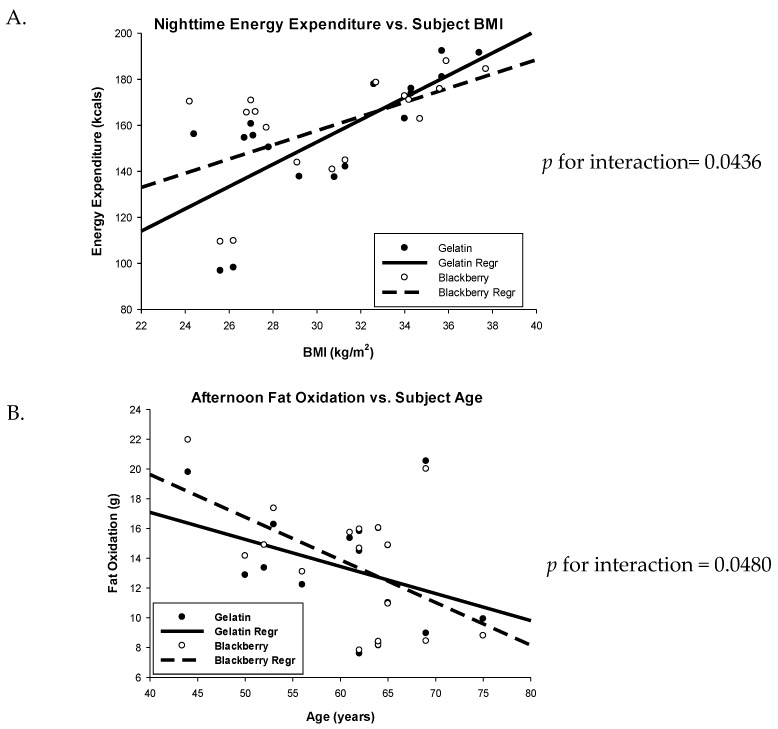

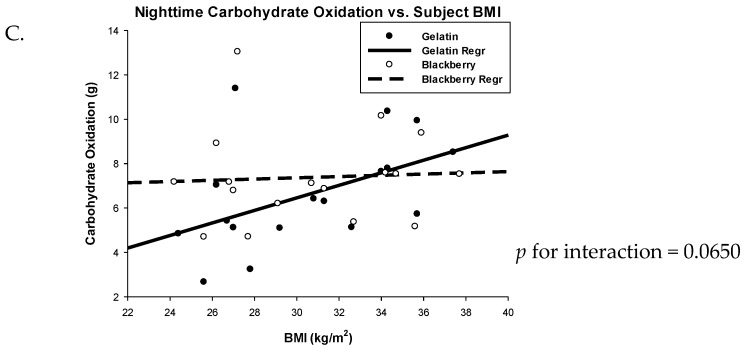
Interactions between treatment and BMI (**A**,**C**) and treatment and age (**B**) for fuel utilization for adult men consuming blackberries vs. gelatin for 7 days. *n* = 17 subjects.

Twenty-four hour calculations for indices of indirect calorimetry represent the shortest time-span during which data was available for every time point across all subjects (21 h and 51 min, no data were extrapolated). The 24-h energy expenditure for the two treatments was very similar (2485 vs. 2439 kcals, BB vs. GEL treatments, respectively, *p* = 0.1055) (Table 4). During the nighttime time isolation, there was a significant interaction between BMI and treatment suggesting that overweight subjects expend more energy while consuming blackberries, where treatment differences are abrogated with some obese subjects and even reversed in others with increasing BMI (Figure 2A, p for interaction = 0.0436). BMI was a significant covariate for energy expenditure for the 24h data set (*p* = 0.04) as well as for 3 of the 5 time isolations (Evening, *p* = 0.023; Morning, *p* = 0.037; Exercise, *p* = 0.029) such that energy expenditure increased with increasing BMI.

### 3.2. Respiratory Quotient and Fuel Oxidation

During BB consumption, there was a significant reduction in RQ for the full 24h period as well as for several time isolations (Table 4), indicating differences in fat and/or carbohydrate oxidation. 24 h RQ was 0.8101 in the BB-fed subjects compared to 0.8171 when they were fed the GEL control (*p* = 0.0402). RQ was also significantly lower during the time isolations of evening (0.8178 vs. 0.8358, BB vs. GEL, respectively, *p* = 0.0063), morning (0.8416 vs. 0.8512, BB vs. GEL, respectively, *p* = 0.0036), and exercise (0.8553 vs. 0.8708, BB vs. GEL, respectively, *p* = 0.0041). These differences in RQ corresponded to a 7% increase in 24h fat oxidation (141 g/day for BB vs. 132 g/day for GEL, *p* = 0.0420), as well as a 14% increase in fat oxidation in the evening (28 g for BB vs. 25 g for GEL, *p* = 0.0069), an 11% increase during the morning (21 vs. 19 g, BB vs. GEL, *p* = 0.0129), and a 12% increase during exercise (9 vs. 8 g, BB vs. GEL, *p* = 0.0044). RQ and fat oxidation were not different for the nighttime and afternoon time isolations. However, in the afternoon, there was a significant interaction between age and treatment suggesting that younger subjects oxidized more fat while consuming BB (8–22 g fat) compared to GEL (8–20 g fat), while this effect was not noted in older subjects (Figure 2B, *p* for interaction = 0.0480). After the GEL treatment, carbohydrate oxidation was 16% greater during the evening (*p* = 0.0061) and 10% greater during exercise (*p* = 0.0497), and demonstrated a trend for increase with GEL over the 24 h period (*p* = 0.0678), but was not different in the morning, afternoon, or nighttime (Table 4). 

BMI was a significant or marginally significant covariate for several outcome variables. BMI was a marginally significant covariate for 24 h fat oxidation (*p* = 0.0516) and afternoon fat oxidation (*p* = 0.0692) where fat oxidized increased with BMI independent of treatment. Similarly, BMI was a significant covariate for exercise CHO oxidation, where CHO oxidized increased with BMI independent of treatment (*p* = 0.0281). In addition, there was a marginal interaction between BMI and treatment for nighttime CHO oxidation, where greater CHO oxidation was observed in lower BMI subjects while administered the BB treatment, however, the diet effect was diminished or even reversed, but to a lesser degree, in obese subjects (Figure 2C, *p* for interaction = 0.0650). These interactions may suggest moderate increases in basal metabolic rate are attributable to an increase in CHO oxidation in overweight but not obese subjects with BB feeding. Furthermore, these differences in subjects’ RQ and subsequent calculations on fat oxidation, either calculated as a 24h average, or reduced to shorter time intervals of post-prandial, basal, or moderate exercise activities, suggest modulation by BBs which may indeed indicate the preferential oxidation of fatty acid substrates. 

### 3.3. Glucose Metabolism and Insulin Sensitivity

**Figure 3:**
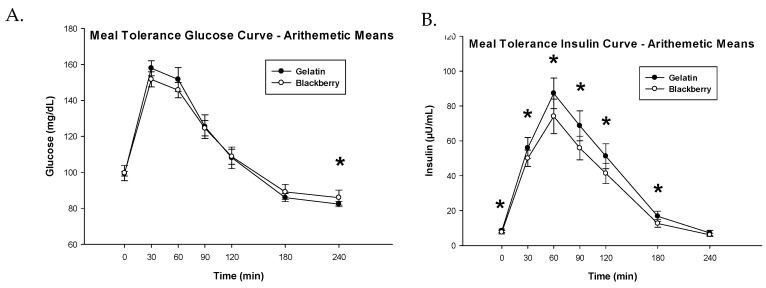
Blood glucose (**A**) and insulin (**B**) concentrations as a function of time after administration of the meal-based glucose tolerance test including either blackberries or gelatin. Asterisks indicate significant differences between the gelatin and blackberry diet treatments, *p* < 0.05. Time-response curves are arithmetic means ± SE. *n* = 24 subjects.

Both the glucose and insulin curves required ln transformation prior to parametric analyses. One subject’s MTT measurements were excluded from analyses due to self-reported noncompliance. The difference between diet treatments for glucose iAUC was not significantly different (3488 vs. 4070 mg·min/dL, BB vs. GEL, respectively, *p* = 0.1151, Figure 3A, Table 5). However, the difference between treatments for insulin iAUC was significant, where subjects had significantly lower iAUC when fed the BB diet compared to GEL control (6485 vs. 8245 µU·min/mL, BB vs. GEL, respectively, *p* = 0.0002, Figure 3B, Table 5). BMI was a significant covariate (*p* = 0.0002) where insulin iAUC increased with increasing BMI. Additionally, a significant sequence effect of the model indicates subjects who ate gelatin and crossed-over to the blackberry treatment had a higher insulin iAUC on average (iAUC = 9589 µU·min/mL) compared to subjects who were assigned the BB diet, then crossed-over onto the GEL treatment (iAUC = 5576 µU·min/mL), *p* = 0.0284. This significant term reveals that there was a potential carryover effect of BBs on insulin sensitivity. However, this effect is only present with the inclusion of two subjects with high residuals, i.e., a sequence effect was not observed and ln transformation of the dataset was not required if the two outliers were removed; with outliers removed, iAUC remained lower with BBs (6681 µU·min/mL) compared to GEL (8232 µU·min/mL), *p* = 0.0067. Conversely, if these data were treated as having a carryover effect and only sequence 1 subjects’ data (gelatin then blackberries) were analyzed, the same conclusion would remain, where subjects had significantly lower insulin iAUC when fed BBs (8528 µU·min/mL) compared to GEL (10841 µU·min/mL), *p* = 0.0143. 

**Figure 4:**
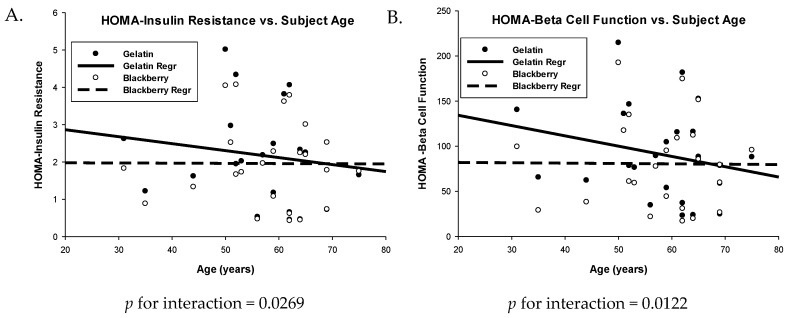
HOMA-IR (**A**) and HOMA-β (**B**) indices for adult men after consuming a diet containing blackberries vs. gelatin for seven days. Scatter plots are model predicted dependent variables vs. age separated by diet treatment. *n* = 24 subjects.

The HOMA-IR dataset required ln transformation, whereas the HOMA-B was normal. There was a significant reduction in HOMA-IR scores when subjects were fed the BB diet (LSmean = 1.5979, backtransformed) compared to the GEL control (LSmean = 1.7630, backtransformed), *p* = 0.0318, Table 5. Further, there was a significant treatment by age interaction (*p* for interaction = 0.0269) which suggested that a reduction in HOMA-IR scores was more prominent in lower age subjects, Figure 4A. BMI was a significant covariate in the model (*p* = 0.0002) where HOMA-IR scores increased with increasing BMI independent of diet treatment. Mirroring the 9% improvement in HOMA-IR was a significant 11% reduction in β-cell function (HOMA-B scores, *p* = 0.0175), where subjects’ scores were 91.2% on average with the GEL control, and 80.3% with the BB diet. Again, there was a significant treatment by age interaction (*p* for interaction = 0.0122) where improvements were less dramatic in older subjects, Figure 4B. BMI was also a significant covariate in the HOMA-B model (*p* = 0.0025), demonstrating the same effect of increased HOMA-B score with increasing BMI. 

**Figure 5:**
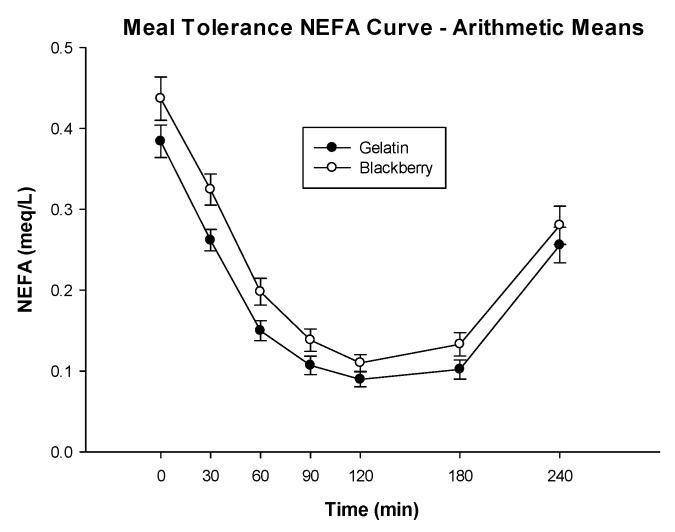
Plasma non-esterified fatty acid concentration as a function of time after administration of the meal-based glucose tolerance test including either blackberries or gelatin. Curves are arithmetic means ± SE. *n* = 24 subjects.

Serum NEFA AUC was significantly higher on average when the subjects were fed the BB diet treatment (LSmean = 48 meq·min/L) compared to when they consumed the GEL control (LSmean = 39 meq·min/L) (*p* = 0.0009), Figure 5. Finally, the analysis for fasting triglycerides required a reciprocal transformation for parametric analysis. There was no significant difference between diet treatments when comparing fasting triglycerides (90 vs. 94 mg/dL, BB vs. GEL, respectively, *p* = 0.2707), Table 5. BMI was a significant covariate in the model which revealed higher fasting triglycerides with increasing BMI. 

## 4. Discussion

Augmentation of lifestyle modifications with micronutrient and phytochemical rich foods as a means to promote weight loss remains an attractive approach for dieticians, physicians, and the consumer. They are relatively inexpensive and consumers have easy access to these “functional” foods when compared to pharmacotherapies or invasive surgeries. In order to improve the chances of increased weight loss, or curbed weight gain, nutritionists must be able to demonstrate which foods promote this activity in order to equip dieticians with the most effective dietary approaches to combat obesity. 

Rodent studies have successfully demonstrated the anti-obesity effects of anthocyanin-rich foods or treatments such as berries and purple corn [8,9,10,15,25,26]. Alongside reductions in weight gain and adiposity, these same studies reported an increase in insulin sensitivity. Human studies using freeze-dried berries or purified anthocyanin extracts have reported less consistent results [27,28,29,30,31,32,33,34,35,36]. One limiting factor of the human research to date has been the lack of control on the diet of the study participants. 

Our study subjects exhibited increased fat oxidation when blackberries were included in the diet. This is in agreement with the one other published study using calorimetry to assess fuel use in humans consuming anthocyanins [37]. In that study, fat oxidation increased 27% when the athletes consumed the black currant extract for seven days compared to placebo during moderate intensity cycling (65% of VO_2max_) [37]. Our findings of increased fat oxidation during a 30-min bout of low intensity treadmill walking with the blackberry treatment support their findings and the concept that anthocyanins can promote fat oxidation during physical activity. Wei et al. [26] observed an increase in skeletal muscle lipoprotein lipase (LPL) activity and lower adipose tissue LPL activity in mice fed C3G, which would be in accordance with increased fat oxidation. How exactly the blackberries are acting to influence substrate oxidation, but less-so energy expenditure requires future work. 

Tea, another flavonoid-rich dietary component, has also been investigated for its ability to influence energy expenditure and substrate oxidation. Rumpler et al. [38] investigated the effects of oolong tea on measures of indirect calorimetry after noting weight loss interventions with oolong tea [39]. Twelve men consumed 1.5 L per day of full-strength oolong tea, a caffeine-matched positive water control, or a placebo water control in a crossover, randomized design for three days with a 23 hr stay in a room-sized calorimetry chamber on the third day. Subjects expended 280 more calories when consuming oolong tea and there was a 12% increase in 24-h fat oxidation when compared to placebo water [39], though these values were not different from those for the caffeine control. Alternatively, Dulloo et al. [40] observed a significant reduction in RQ, and an increase in EE in healthy, sedentary men receiving a single daily dose of green tea extract compared to a caffeine placebo. In addition, a study by Rudelle et al. [41] reported a 4.6% increase in EE after consumption of a beverage containing green tea catechins, caffeine, and calcium, though no effect was observed on specific substrate oxidation. In contrast to the null findings on substrate oxidation by Rudelle et al., Gahreman et al. [42] showed that green tea extract administered along with sprinting exercises increased fat oxidation in healthy weight, untrained young females both before and shortly after exercise by increments of 24% and 29%, respectively. In another study, 12 overweight men showed an increase in postprandial fat oxidation, measured using a ventilated hood, after consuming a dose of the tea flavonoid EGCG for three days compared to placebo [43], though a higher dose of the tea flavonoid did not result in the same effect. These human studies involving tea products may act through different mechanisms compared to the blackberry intervention of this study, as they show more pronounced effects on energy expenditure with less evidence on affecting the partitioning of a fuel substrate, whereas the opposite appears to be the case with the blackberry treatment in this study. One obvious difference in comparing these interventions is the caffeine content of tea. 

Our study demonstrated an age dependent improvement in insulin sensitivity with blackberry intake, with younger subjects being most responsive. Other studies have also described an anti-diabetic effect of berry consumption [32,33,44,45,46] and have been described in two meta-analyses [28,47]. One possible explanation for the insulin sensitizing effect of berries is the activation of adenosine monophosphate protein kinase across several tissues involved in glucoregulation [16,48]. Furthermore, amelioration of fatty acid overload may be possible by the beige-adipocyte phenotype invoked by cyanidin-3-glucoside, thereby improving insulin signaling [49]. Other mechanistic work highlights the potential to inhibit adipogenic enzymes of the liver as well as reduce the absorption of glucose from the gut [8,50]. Thus, berry anthocyanins likely invoke positive effects in myriad pathways related to glucoregulatory control. An unexpected finding from this study was the higher serum NEFA AUC with the BB treatment during the MTT, which occurred in parallel to the significant reduction in insulin AUC, despite no change in the glucose curve. Higher circulating NEFAs are classically perceived as an instigator in reduced insulin sensitivity and increased hepatic glucose output [51,52]. NEFA overload from the visceral adipose depot is believed to complicate the metabolic syndrome [53]. However, a recent tracer study modeling NEFA kinetics in obese men and women reported the dual-action of insulin in the regulation of fatty acid release and uptake by peripheral tissues [54]. One possible explanation for our observation may be that the increase in insulin sensitivity (reduced insulin secretion during the MTT) with BBs may have yielded higher circulating NEFAs due to less constraints on these insulin-dependent regulatory mechanisms. Alternatively, as described by Lambert and Parks, berries may have an effect on the “spillover” pathway of lipoprotein clearance [55]. Fatty acid “spillover” refers to the release of fatty acids into the blood stream after cleavage from chylomicron triglycerides by LPL. Thus, the increase in NEFAs may be related to Wei et al.’s [26] finding that C3G activates LPL associated with skeletal muscle.

One limitation of this study was that the treatments could not be blinded, since we were using whole foods. Another limitation of this study was that, while the total carbohydrate content of the two meals was matched extremely closely (106 g CHO for the gelatin/pancake meal vs. 108 g CHO for the blackberry/pancake meal), the sugar content of the two meals used for the glucose tolerance test were slightly different (81.9 g sugar for the gelatin/pancake meal vs. 70.9 g sugar for the blackberry/pancake meal). Since the insulin response would be driven by the total carbohydrate load rather than just sugar, and since the glucose responses were not different between treatments, the slight difference in sugar content of the treatment and control is likely not the cause of the improved insulin response when subjects were consuming berries.

## 5. Conclusions

In conclusion, BB feeding significantly increased fat oxidation and insulin sensitivity in overweight or obese men fed a high-fat diet including 600 g blackberries per day for one week compared to an energy matched control treatment in a randomized, crossover study. This study offers translation of the anti-obesity effects of berries observed in rodent models to humans by means of indirect calorimetry. Further studies will be useful to clarify the findings and potential mechanisms of action.

## Figures and Tables

**Table 1:** Flavonoid content of blackberries provided to participants for 7 days as part of a controlled diet.

Flavonoid	Per 300 g Serving	Per 600 g Daily Dose
Cyanidin 3-glucoside (mg)	180.7	361.3
Catechin (mg)	32.9	65.8
Epicatechin (mg)	200.9	401.7
Kaempferol glucoside (mg)	283.7	567.3
Quercetin glucoside (mg)	39.7	79.4
Total measured flavonoids (mg)	737.9	1475.8

**Table 2:** Macronutrient content of components of the meal-based glucose tolerance test provided at the end of the 7-day controlled feeding periods.

	Quantity (g)	Protein (g)	CHO ^1^ (g)	Sugar (g)	Fat (g)	Energy (kcal)
Blackberry treatment						
Waffles	56.5	3.3	20.5	2.4	3.7	128.5
Pancake Syrup	60	0	40.2	36.3	0	160.8
Blackberries, unsweetened	302	3.6	47.3	32.2	1.3	215.3
Total	418.5	6.9	108	70.9	5	504.6
Gelatin treatment						
Waffles	56.5	3.3	20.5	2.4	3.7	128.5
Pancake Syrup	60	0	40.2	36.3	0	160.8
Gelatin, strawberry	273	2.8	45.5	43.2	0	193.2
Total	389.5	6.1	106.2	81.9	3.7	482.5

^1^ CHO (carbohydrate) accounts for sugar and non-sugar carbohydrate.

**Table 3:** Baseline subject characteristics reported for each dataset.

	Glucose Tolerance (means ± SE, *n* = 24)	Calorimetry (means ± SE, *n* = 17)	*p* ^1^
Age (years)	57.8 ± 2.1	61 ± 1.9	0.2868
Weight (kg)	95.7 ± 2.6	94.2 ± 3.3	0.7319
BMI (kg/m^2^)	30.6 ± 0.8	30.7 ± 1	0.9463
Total Cholesterol (mg/dL)	186.9 ± 8.7	175.8 ± 11.7	0.4426
LDL Cholesterol (mg/dL)	116 ± 7.6	104.7 ± 9.8	0.3589
HDL Cholesterol (mg/dL)	46.1 ± 2.2	47.5 ± 3	0.7007
Triglycerides (mg/dL)	124.1 ± 11.6	118.2 ± 12	0.7338
Systolic BP (mm Hg)	123.3 ± 3	123.1 ± 3.5	0.9629
Diastolic BP (mm Hg)	75.6 ± 1.7	76.2 ± 1.9	0.8202
Glucose (mg/dL)	98.6 ± 1.4	97.6 ± 1.4	0.6403

^1^*p*-Values for two-sided *t*-tests.

**Table 4:** Respiratory quotient, energy expenditure, and substrate oxidation after consumption of blackberries or gelatin for 7 days.

	Gelatin	Blackberry		
	LSmean (95% CI)	LSmean (95% CI)	Difference (95% CI)	*p* ^1^
RQ (CO_2_/O_2_)				
24 h	0.8171 (0.8079, 0.8262)	0.8101 (0.801, 0.8192)	0.0069 (0.0004, 0.0135)	0.0402
Evening	0.8358 (0.8229, 0.8486)	0.8178 (0.805, 0.8306)	0.018 (0.00594, 0.0301)	0.0063
Nighttime	0.7928 (0.779, 0.8066)	0.796 (0.7822, 0.8098)	−0.0031 (−0.0135, 0.0072)	0.5289
Morning	0.8512 (0.8403, 0.862)	0.8416 (0.8308, 0.8525)	0.0095 (0.0036, 0.0155)	0.0036
Afternoon	0.8135 (0.8003, 0.8267)	0.8098 (0.7966, 0.823)	0.0037 (−0.0049, 0.0123)	0.3806
Exercise	0.8708 (0.8615, 0.8801)	0.8553 (0.846, 0.8646)	0.0155 (0.0057, 0.0253)	0.0041
Fat Oxidation (g)				
24 h	132.38 (117.22, 147.53)	140.84 (125.69, 156)	−8.47 (−16.59, −0.35)	0.042
Evening	24.6 (21.34, 27.85)	27.98 (24.73, 31.24)	−3.39 (−5.70, −1.07)	0.0069
Nighttime	9.49 (8.34, 10.63)	9.48 (8.34, 10.63)	0.01 (−1.61, 1.62)	0.9935
Morning	19.35 (16.58, 22.12)	21.30 (18.53, 24.07)	−1.95 (−3.43, −0.47)	0.0129
Afternoon	13.30 (11.37, 15.23)	13.63 (11.70, 15.56)	−0.33 (−1.08, 0.42)	0.3671
Exercise	8.32 (7.06, 9.59)	9.35 (8.08, 10.62)	−1.03 (−1.68, −0.37)	0.0044
Carbohydrate Oxidation (g)				
24 h	182.71 (166.19, 199.24)	172.95 (156.42, 189.47)	9.77 (−0.80, 20.33)	0.0678
Evening	41.56 (37.14, 45.98)	35.87 (31.45, 40.29)	5.69 (1.87, 9.51)	0.0061
Nighttime	6.6 (5.11, 8.09)	7.35 (5.86, 8.83)	−0.75 (−1.86, 0.37)	0.1727
Morning	43.11 (39.80, 46.42)	41.13 (37.82, 44.44)	1.98 (−0.85, 4.81)	0.1567
Afternoon	15.02 (12.1, 17.94)	15.45 (12.53, 18.37)	−0.43 (−2.91, 2.05)	0.7185
Exercise	25.71 (23.07, 28.35)	23.46 (20.82, 26.10)	2.24 (0.00, 4.47)	0.0497
Energy Expenditure (kcals)				
24 h	2438.5 (2254.16, 2622.83)	2485.4 (2301.06, 2669.75)	−46.91 (−104.86, 11.05)	0.1055
Evening	483.38 (449.84, 516.91)	493.29 (459.75, 526.82)	−9.91 (−27.96, 8.15)	0.2616
Nighttime	155.71 (143.59, 167.83)	159.63 (147.51, 171.75)	−3.92 (−10.72, 2.88)	0.2384
Morning	439.99 (406.11, 473.88)	451.67 (417.79, 485.56)	−11.68 (−30.32, 6.96)	0.2028
Afternoon	224.94 (203.72, 246.15)	230.4 (209.18, 251.61)	−5.46 (−17.94, 7.02)	0.3674
Exercise	196.37 (175.68, 217.06)	197.36 (176.67, 218.05)	−1.00 (−7.98, 5.99)	0.7667

^1^*p*-Values for group-wise differences. *N* = 17 subjects.

**Table 5:** Biochemical measures in blood after consumption of blackberries or gelatin for seven days.

	Gelatin	Blackberry		
	LSmean (95% CI)	LSmean (95% CI)	Difference (95% CI)	*p* ^3^
Glucose iAUC (mg·min/dL) ^1^	8.31 (8.05, 8.57)	8.16 (7.90, 8.41)	0.15 (−0.04, 0.35)	0.1151
Insulin iAUC (µU·min/mL) ^1^	9.02 (8.77, 9.26)	8.78 (8.53, 9.02)	0.24 (0.13, 0.35)	0.0002
HOMA-IR ^1^	0.57 (0.34, 0.80)	0.47 (0.24, 0.70)	0.10 (0.01, 0.19)	0.0318
HOMA-β	91.24 (72.22, 110.26)	80.32 (61.30, 99.34)	10.93 (2.11, 19.75)	0.0175
NEFA AUC (meq·min/L)	39.13 (34.09, 44.17)	47.67 (42.63, 52.71)	−8.54 (−13.15, −3.92)	0.0009
Fasting Triglycerides(mg/dL) ^2^	0.0107 (0.009, 0.0123)	0.0111 (0.0094, 0.0127)	−0.0004 (−0.0011, 0.0003)	0.2412

^1^ Dataset is ln transformed. ^2^ Dataset is reciprocal transformed. ^3^ *p*-Values for group-wise differences. *n* = 4 subjects.

## References

[1] Ogden C.L., Carroll M.D., Kit B.K., Flegal K.M. (2014). Prevalence of childhood and adult obesity in the united states, 2011–2012. JAMA.

[2] Centers for Disease Control, Department of Health and Human Services Adult Obesity Causes & Consequences. https://www.cdc.gov/obesity/adult/causes.html.

[3] Dunkley A.J., Charles K., Gray L.J., Camosso-Stefinovic J., Davies M.J., Khunti K. (2012). Effectiveness of interventions for reducing diabetes and cardiovascular disease risk in people with metabolic syndrome: Systematic review and mixed treatment comparison meta-analysis. Diabetes Obes. Metab..

[4] Dauchet L., Amouyel P., Hercberg S., Dallongeville J. (2006). Fruit and vegetable consumption and risk of coronary heart disease: A meta-analysis of cohort studies. J. Nutr..

[5] Wang X., Ouyang Y.Y., Liu J., Zhu M.M., Zhao G., Bao W., Hu F.B. (2014). Fruit and vegetable consumption and mortality from all causes, cardiovascular disease, and cancer: Systematic review and dose-response meta-analysis of prospective cohort studies. Br. Med. J..

[6] Tresserra-Rimbau A., Rimm E.B., Medina-Remon A., Martinez-Gonzalez M.A., Lopez-Sabater M.C., Covas M.I., Corella D., Salas-Salvado J., Gomez-Gracia E., Lapetra J. (2014). Polyphenol intake and mortality risk: A re-analysis of the predimed trial. BMC Med..

[7] Nile S.H., Park S.W. (2014). Edible berries: Bioactive components and their effect on human health. Nutrition.

[8] Tsuda T., Horio F., Uchida K., Aoki H., Osawa T. (2003). Dietary cyanidin 3-o-beta-d-glucoside-rich purple corn color prevents obesity and ameliorates hyperglycemia in mice. J. Nutr..

[9] Farrell N.J., Norris G.H., Ryan J., Porter C.M., Jiang C., Blesso C.N. (2015). Black elderberry extract attenuates inflammation and metabolic dysfunction in diet-induced obese mice. Br. J. Nutr..

[10] Heyman L., Axling U., Blanco N., Sterner O., Holm C., Berger K. (2014). Evaluation of beneficial metabolic effects of berries in high-fat fed c57bl/6j mice. J. Nutr. Metab..

[11] Prior R.L., Wilkes S., Rogers T., Khanal R.C., Wu X.L., Hager T.J., Hager A., Howard L. (2010). Dietary black raspberry anthocyanins do not alter development of obesity in mice fed an obesogenic high-fat diet. J. Agric. Food Chem..

[12] Prior R.L., Wilkes S.E., Rogers T.R., Khanal R.C., Wu X.L., Howard L.R. (2010). Purified blueberry anthocyanins and blueberry juice alter development of obesity in mice fed an obesogenic high-fat diet. J. Agric. Food Chem..

[13] Prior R.L., Wu X.L., Gu L.W., Hager T.J., Hager A., Howard L.R. (2008). Whole berries versus berry anthocyanins: Interactions with dietary fat levels in the c57bl/6j mouse model of obesity. J. Agric. Food Chem..

[14] Seymour E.M., Singer A.A.M., Kirakosyan A., Urcuyo-Llanes D.E., Kaufman P.B., Bolling S.F. (2008). Altered hyperlipidemia, hepatic steatosis, and hepatic peroxisome proliferator-activated receptors in rats with intake of tart cherry. J. Med. Food.

[15] Seymour E.M., Tanone I.I., Urcuyo-Llanes D.E., Lewis S.K., Kirakosyan A., Kondoleon M.G., Kaufman P.B., Bolling S.F. (2011). Blueberry intake alters skeletal muscle and adipose tissue peroxisome proliferator-activated receptor activity and reduces insulin resistance in obese rats. J. Med. Food.

[16] Takikawa M., Inoue S., Horio F., Tsuda T. (2010). Dietary anthocyanin-rich bilberry extract ameliorates hyperglycemia and insulin sensitivity via activation of amp-activated protein kinase in diabetic mice. J. Nutr..

[17] Webb P. (1986). 24-hour energy expenditure and the menstrual cycle. Am. J. Clin. Nutr..

[18] (2015). 2015–2020 Dietary Guidelines for Americans.

[19] Redan B.W., Albaugh G.P., Charron C.S., Novotny J.A., Ferruzzi M.G. (2017). Adaptation in caco-2 human intestinal cell differentiation and phenolic transport with chronic exposure to blackberry (rubus sp.) extract. J. Agric. Food Chem..

[20] Seale J.L., Rumpler W.V., Moe P.W. (1991). Description of a direct-indirect room-sized calorimeter. Am. J. Physiol..

[21] Gribok A., Hoyt R., Buller M., Rumpler W. (2013). On the accuracy of instantaneous gas exchange rates, energy expenditure and respiratory quotient calculations obtained from indirect whole room calorimetry. Physiol. Meas..

[22] Gribok A., Leger J.L., Stevens M., Hoyt R., Buller M., Rumpler W. (2016). Measuring the short-term substrate utilization response to high-carbohydrate and high-fat meals in the whole-body indirect calorimeter. Physiol. Rep..

[23] Livesey G., Elia M. (1988). Estimation of energy expenditure, net carbohydrate utilization, and net fat oxidation and synthesis by indirect calorimetry: Evaluation of errors with special reference to the detailed composition of fuels. Am. J. Clin. Nutr..

[24] Wallace T.M., Levy J.C., Matthews D.R. (2004). Use and abuse of homa modeling. Diabetes Care.

[25] Bhaswant M., Fanning K., Netzel M., Mathai M.L., Panchal S.K., Brown L. (2015). Cyanidin 3-glucoside improves diet-induced metabolic syndrome in rats. Pharmacol. Res..

[26] Wei X., Wang D., Yang Y., Xia M., Li D., Li G., Zhu Y., Xiao Y., Ling W. (2011). Cyanidin-3-o-beta-glucoside improves obesity and triglyceride metabolism in kk-ay mice by regulating lipoprotein lipase activity. J. Sci. Food Agric..

[27] Huang H.H., Chen G.Z., Liao D., Zhu Y.K., Xue X.Y. (2016). Effects of berries consumption on cardiovascular risk factors: A meta-analysis with trial sequential analysis of randomized controlled trials. Sci. Rep. UK.

[28] Guo X., Yang B., Tan J., Jiang J., Li D. (2016). Associations of dietary intakes of anthocyanins and berry fruits with risk of type 2 diabetes mellitus: A systematic review and meta-analysis of prospective cohort studies. Eur J. Clin. Nutr..

[29] Qin Y., Xia M., Ma J., Hao Y., Liu J., Mou H., Cao L., Ling W. (2009). Anthocyanin supplementation improves serum ldl- and hdl-cholesterol concentrations associated with the inhibition of cholesteryl ester transfer protein in dyslipidemic subjects. Am. J. Clin. Nutr..

[30] Zhu Y., Xia M., Yang Y., Liu F., Li Z., Hao Y., Mi M., Jin T., Ling W. (2011). Purified anthocyanin supplementation improves endothelial function via no-cgmp activation in hypercholesterolemic individuals. Clin. Chem..

[31] Zhu Y., Huang X., Zhang Y., Wang Y., Liu Y., Sun R., Xia M. (2014). Anthocyanin supplementation improves hdl-associated paraoxonase 1 activity and enhances cholesterol efflux capacity in subjects with hypercholesterolemia. J. Clin. Endocrinol. Metab..

[32] Hoggard N., Cruickshank M., Moar K.M., Bestwick C., Holst J.J., Russell W., Horgan G. (2013). A single supplement of a standardised bilberry (*Vaccinium myrtillus* L.) extract (36% wet weight anthocyanins) modifies glycaemic response in individuals with type 2 diabetes controlled by diet and lifestyle. J. Nutr. Sci..

[33] Stull A.J., Cash K.C., Johnson W.D., Champagne C.M., Cefalu W.T. (2010). Bioactives in blueberries improve insulin sensitivity in obese, insulin-resistant men and women. J. Nutr..

[34] Stull A.J., Cash K.C., Champagne C.M., Gupta A.K., Boston R., Beyl R.A., Johnson W.D., Cefalu W.T. (2015). Blueberries improve endothelial function, but not blood pressure, in adults with metabolic syndrome: A randomized, double-blind, placebo-controlled clinical trial. Nutrients.

[35] Basu A., Fu D.X., Wilkinson M., Simmons B., Wu M.Y., Betts N.M., Du M., Lyons T.J. (2010). Strawberries decrease atherosclerotic markers in subjects with metabolic syndrome. Nutr. Res..

[36] Basu A., Du M., Leyva M.J., Sanchez K., Betts N.M., Wu M., Aston C.E., Lyons T.J. (2010). Blueberries decrease cardiovascular risk factors in obese men and women with metabolic syndrome. J. Nutr..

[37] Cook M.D., Myers S.D., Blacker S.D., Willems M.E. (2015). New zealand blackcurrant extract improves cycling performance and fat oxidation in cyclists. Eur. J. Appl. Physiol..

[38] Rumpler W., Seale J., Clevidence B., Judd J., Wiley E., Yamamoto S., Komatsu T., Sawaki T., Ishikura Y., Hosoda K. (2001). Oolong tea increases metabolic rate and fat oxidation in men. J. Nutr..

[39] Chen W., Yang Z., Hosoda K., Chen L., Lin B., Kimura J., Matsui Y., Matsui K. (1998). Clinical efficacy of oolong tea on anti-simple obesity. Jpn. J. Clin. Nutr..

[40] Dulloo A.G., Duret C., Rohrer D., Girardier L., Mensi N., Fathi M., Chantre P., Vandermander J. (1999). Efficacy of a green tea extract rich in catechin polyphenols and caffeine in increasing 24-h energy expenditure and fat oxidation in humans. Am. J. Clin. Nutr..

[41] Rudelle S., Ferruzzi M.G., Cristiani I., Moulin J., Mace K., Acheson K.J., Tappy L. (2007). Effect of a thermogenic beverage on 24-hour energy metabolism in humans. Obesity.

[42] Gahreman D., Wang R., Boutcher Y., Boutcher S. (2015). Green tea, intermittent sprinting exercise, and fat oxidation. Nutrients.

[43] Thielecke F., Rahn G., Bohnke J., Adams F., Birkenfeld A.L., Jordan J., Boschmann M. (2010). Epigallocatechin-3-gallate and postprandial fat oxidation in overweight/obese male volunteers: A pilot study. Eur. J. Clin. Nutr..

[44] Liu Y., Li D., Zhang Y., Sun R., Xia M. (2014). Anthocyanin increases adiponectin secretion and protects against diabetes-related endothelial dysfunction. Am. J. Physiol. Endocrinol. Metab..

[45] Park E., Edirisinghe I., Wei H., Vijayakumar L.P., Banaszewski K., Cappozzo J.C., Burton-Freeman B. (2016). A dose-response evaluation of freeze-dried strawberries independent of fiber content on metabolic indices in abdominally obese individuals with insulin resistance in a randomized, single-blinded, diet-controlled crossover trial. Mol. Nutr. Food Res..

[46] Moazen S., Amani R., Homayouni Rad A., Shahbazian H., Ahmadi K., Taha Jalali M. (2013). Effects of freeze-dried strawberry supplementation on metabolic biomarkers of atherosclerosis in subjects with type 2 diabetes: A randomized double-blind controlled trial. Ann. Nutr. Metab..

[47] Wang P.Y., Fang J.C., Gao Z.H., Zhang C., Xie S.Y. (2016). Higher intake of fruits, vegetables or their fiber reduces the risk of type 2 diabetes: A meta-analysis. J. Diabetes Investig..

[48] Tsuda T. (2016). Recent progress in anti-obesity and anti-diabetes effect of berries. Antioxidants.

[49] Matsukawa T., Villareal M.O., Motojima H., Isoda H. (2016). Increasing camp levels of preadipocytes by cyanidin-3-glucoside treatment induces the formation of beige phenotypes in 3t3-l1 adipocytes. J. Nutr. Biochem..

[50] Matsui T., Ueda T., Oki T., Sugita K., Terahara N., Matsumoto K. (2001). Alpha-glucosidase inhibitory action of natural acylated anthocyanins. 1. Survey of natural pigments with potent inhibitory activity. J. Agric. Food Chem..

[51] Stefan N., Stumvoll M., Bogardus C., Tataranni P.A. (2003). Elevated plasma nonesterified fatty acids are associated with deterioration of acute insulin response in igt but not ngt. Am. J. Physiol. Endocrinol. Metab..

[52] Walker M., Agius L., Orskov H., Alberti K.G. (1993). Peripheral and hepatic insulin sensitivity in non-insulin-dependent diabetes mellitus: Effect of nonesterified fatty acids. Metabolism.

[53] Grundy S.M. (2011). Atlas of Atherosclerosis and Metabolic Syndrome.

[54] Ramos-Roman M.A., Lapidot S.A., Phair R.D., Parks E.J. (2012). Insulin activation of plasma nonesterified fatty acid uptake in metabolic syndrome. Arterioscler. Thromb. Vasc. Biol..

[55] Lambert J.E., Parks E.J. (2012). Postprandial metabolism of meal triglyceride in humans. Biochim. Biophys. Acta..

